# Wild Gorillas Handy with a Stick

**DOI:** 10.1371/journal.pbio.0030385

**Published:** 2005-10-01

When chimpanzees were first seen using tools in Liberia in 1951, little was known about great ape behavior in the wild. The sighting was published as a short note by Harry Beatty in the *Journal of Mammalogy* (recounted by primatologist Frans de Waal in *The Ape and the Sushi Master*). After spotting a shell mound, Beatty saw a chimp “come ambling round a bend bearing an armful of dried palm nuts,” sit down beside a rock, select a nut, place it on a flat rock surface, and pound it with another rock to extract the meat. Thirty years passed before primatologists would describe the same behavior in chimps in Guinea. By then it was clear that nonhuman primates used objects for many tasks, from intimidating rivals and predators to taking leaf sponge baths and processing food. Still, the notion that tool use was not the sole province of human intelligence was difficult for some to accept.

Though chimps, bonobos, orangutans, and gorillas all use tools in captivity, often imitating behaviors of their keepers, tool use had not been observed for gorillas in the wild. (Chimps famously use sticks to fish for termites and rocks to crack nuts in the wild; orangutans use sticks to extract seeds from the spiny, sharp husk of Neesia fruits in the wild, and bonobos use various tools, including moss as sponges and leafy twigs to swat away bees.) That no one has ever observed wild gorillas using tools—despite decades of intensive study—prompted scientists to speculate that gorillas lost the skill because they didn't need it. The largest of the great apes, gorillas can gnash nuts between their teeth and simply smash termite mounds to release their denizens. [Fig pbio-0030385-g001]

**Figure pbio-0030385-g001:**
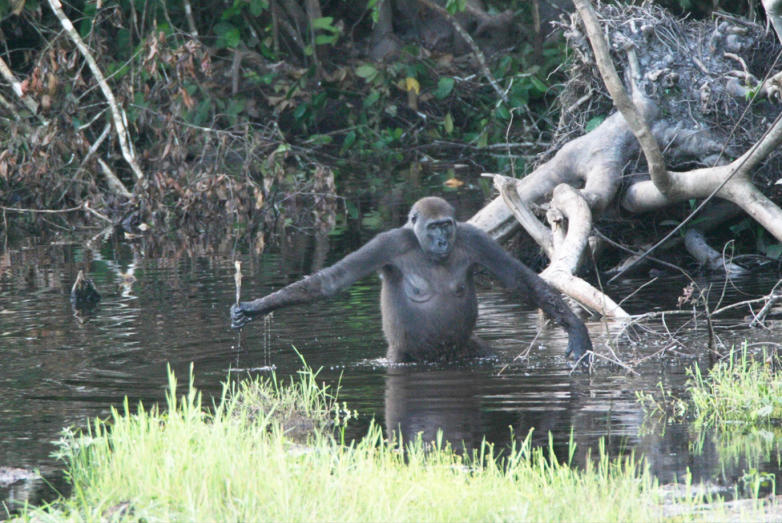
A first time for everything: This adult female gorilla in Nouabalé-Ndoki National Park, northern Congo, uses a branch as a walking stick to gauge the water's depth, proving that gorillas use tools too

But now, as part of an ongoing study of western gorillas in Nouabalé-Ndoki National Park in the Republic of Congo, Thomas Breuer, Mireille Ndoundou-Hockemba, and Vicki Fishlock reveal that gorillas are just as resourceful as the other great apes. From an observation platform at Mbeli Bai, a swampy forest clearing that gorillas frequently visit to forage, Breuer et al. observed an adult female gorilla named Leah (a member of a long-studied gorilla group) at the edge of a pool of water, “looking intently at the water in front of her.” Leah walked upright into the water, but stopped and returned to the edge when the water reached her waist. She then walked back into the water, grabbed a branch in front of her, detached it, and, grasping it firmly, repeatedly jabbed the water in front of her with the end of the branch, “apparently using it to test the water depth or substrate stability.” She continued walking across the pool, branch in hand, “using it as a walking stick for postural support.”

In a separate incident, a second adult female named Efi (who belonged to a different group) emerged from the forest and broke off the thin, long trunk from a dead shrub with both hands. She then jammed the trunk into the ground and grasped it with her left hand while manipulating food with her right hand. Efi then placed the trunk on the swampy ground in front of her, and, walking upright, used it as a bridge to cross the muck.

That tool use was seen in lowland gorillas and not mountain gorillas—the most intensively studied gorillas—may reflect the different ecological challenges facing the two populations, supporting the notion that ecology influences the evolution of novel tool use. Though Mbeli Bai gorillas often use intact branches to help them get around, the authors explain, these are the first observations of wild gorillas using detached objects as tools. However the gorillas acquired their skills, these observations suggest that the intelligence required for tool use evolved before the gorilla lineage split off from humans and the other great apes—providing further evidence that intelligence is not unique to humans. —*Liza Gross*

